# Synthesis and Liquid Crystalline Properties of New Diols Containing Azomethine Groups

**DOI:** 10.3390/molecules15053260

**Published:** 2010-04-30

**Authors:** Issam Ahmed Mohammed, Govindarajan Sankar, Melati Khairuddean, Abu Bakar Mohamad

**Affiliations:** 1 School of Industrial Technology, University Sains Malaysia, 11800 Penang, Malaysia; 2 School of Chemical Sciences, University Sains Malaysia, 11800 Penang, Malaysia

**Keywords:** thermotropic liquid crystal, nematic phase, diols, azomethine

## Abstract

A series of new mesogenic azomethine diols were successfully synthesized by condensation reactions between various chloroalkanols and *N,N'*-bis(4-hydroxy)-benzylidene-*o*-toluidine (**1**). The structures of these compounds were confirmed by CHN, FT-IR, ^1^H-NMR, and ^13^C-NMR spectrophotometer. Their thermotropic liquid crystalline behavior was studied using differential scanning calorimetry (DSC) and polarizing optical microscope (POM). 4,4'-di(4-Hydroxybutoxy)-*N*-benzylidine-*o*-tolidine (**2a**) does not exhibit liquid crystalline properties. A nematic texture was observed for mesogenic diols **2b,** and **2d**, whereas the diol **2c** exhibits a smectic mesophase. The increase of terminal alkyl chain in these mesogenic diols leads to a decrease in the transition temperature.

## 1. Introduction

Organic compounds exhibit thermotropic liquid crystalline (LC) properties when the molecules possess rod- or disc-like structure. The stabilization and polarizability are two of the most important factors in liquid crystal devices. Researchers have discovered that small molecules are also capable of exerting a significant impact on the molecular polarizability as well as the stabilization in the liquid crystalline compounds. Azomethine (CH=N) linkages present in the backbone provide an attractive class of high performance material. High thermal stability, excellent mechanical strength, and good optoelectronic properties are also attributed to the resonance stabilization of the Schiff’s base unit [1]. However, these small molecules have certain drawbacks such as poor solubility that minimize their practical applications. 

Small molecules (in this case, liquid crystal monomers) with geometrical anisotropy and high polarizability often exhibit one or more liquid crystalline phases besides the crystalline and isotropic phases [2]. Therefore, a terminal functional group such as a hydroxyl group is employed in order to enhance these features. One of the typical examples is the study on the effect of lateral hydroxyl groups on the mesomophism of azobenzene derivatives [3]. To our best knowledge, limited work has been performed on the compounds containing azomethine linkages and hydroxyls as the end group. With this background we decided to synthesize a series of novel *N,N'* bis(4-hydroxyl)benzylidine-*o*-tolidine derivatives and to investigate the liquid crystalline properties of the new diols.

## 2. Result and Discussion

### 2.1. Synthesis and characterization 

**Scheme 1:**
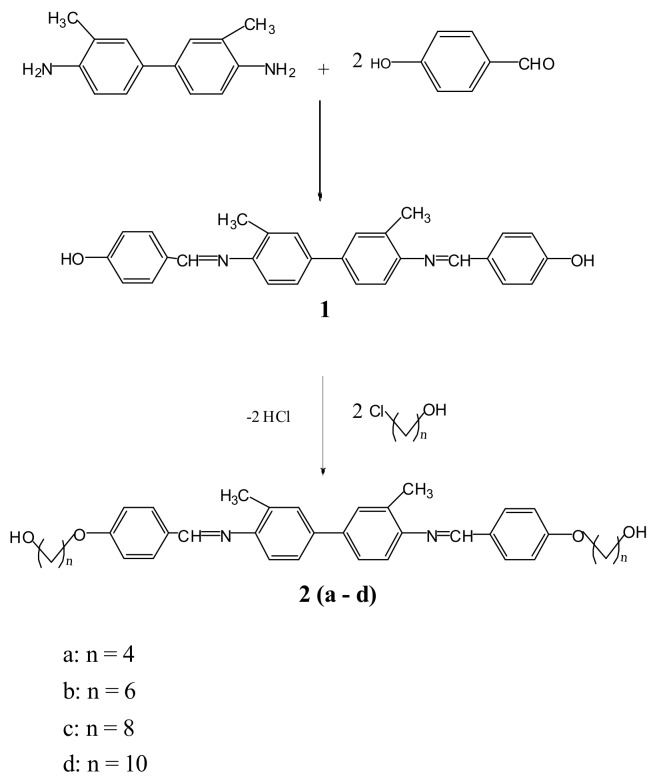
Synthesis of mesogenic diols **2 (a–d**).

**Figure 1:**
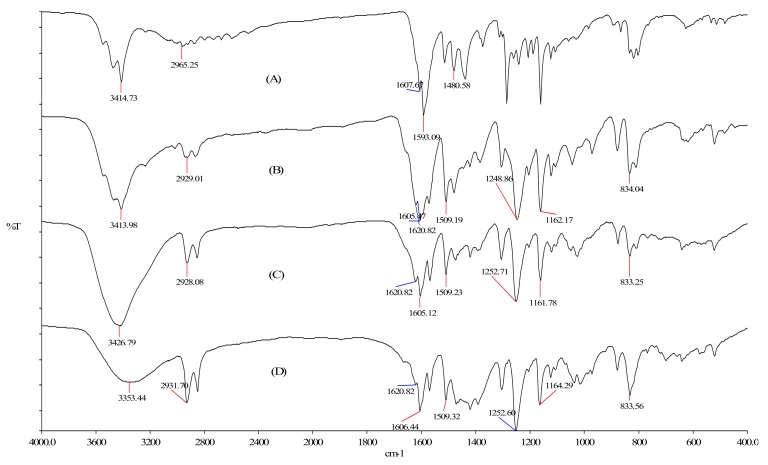
The FT-IR spectra of (A) bisphenol **1** (B) diol **2a,** (C) diol **2b** and (D) diol **2d**.

Preparation of diols **2(a–d**) is explained in Scheme 1. The structures of bisphenol **1** and the diols **2(a–d**) were confirmed by elemental analysis (CHN), FT-IR, ^1^H-NMR and ^13^C-NMR spectrophotometry. Figure **1** shows four different IR spectra representing diols **1**, **2a**, **2b** and **2d**. In the obtained FT-IR spectra the disappearance of the NH_2_ and carbonyl absorption bands at (3,336–3,472) cm^-1^and 1,667 cm^-1^ and the appearance of characteristic absorption bands at 1,620 cm^-1^ assigned to the presence of a -CH=N bond could be observed. This correlates with the data obtained by Issam [4] and So *et al.* [5], where the absorption band for -CH=N group is around 1,619 cm^-1^. The sharp absorption band at (3,350**–**3,414) cm^-1^ is attributed to the -OH group [6,7]. Absorption bands at 1,480 cm^-1^ and 1,592 cm^-1^, which is corresponds to aromatic –C=C–, indicates that an aromatic ring exists in the synthesized compound. The FT-IR data for the diols **2(a–d**) and the reactants are given in Experimental section. 

**Figure 2:**
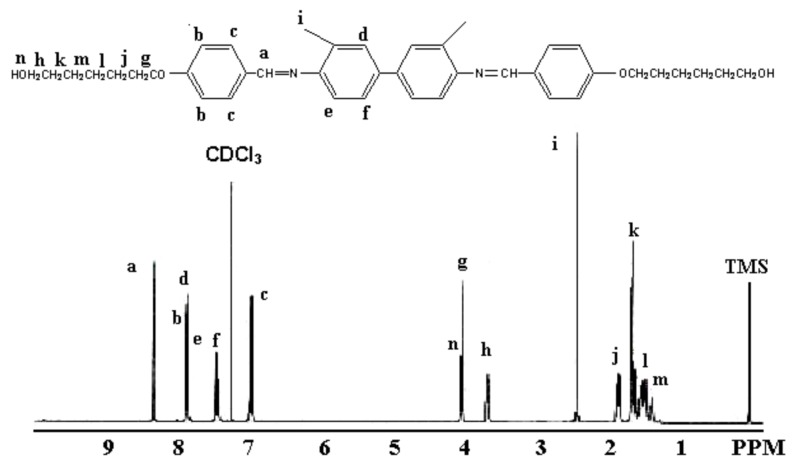
^1^H-NMR spectrum of diol 2b.

**Figure 3:**
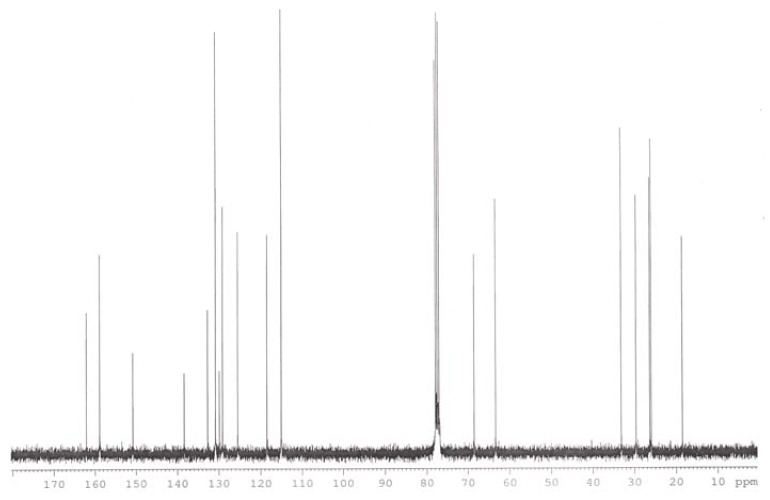
^13^C-NMR spectrum of diol 2b.

Figure 2 and Figure 3 show typical examples of ^1^H-NMR and ^13^C-NMR spectra, corresponding to diol **2b**. In the ^1^H-NMR spectrum, the methylene protons in (HO-CH_2_-) and (Ph-O-CH_2_-) appear at 3.57 ppm and 4.05 ppm, respectively. This is similar to the data that obtained by Stamatoiu *et al.* [8]. The singlet peak at 8.65 ppm is assigned to the azomethine (-CH=N-) group [9]. The azomethine group appeared at higher field because the nitrogen atom is bonded to an aromatic ring and the nitrogen has a lone pair of electrons which enables it to delocalize into the aromatic ring. The bands of the -OH protons are not detected due to overlap with C-H protons. However, we further confirm formation of hydroxyl group by FT-IR which indicates the absorption band at 3,420 cm^-1^. ^1^H-NMR data for the diols **2(a–d**) are given in the Experimental section and the properties are summarized in Table 1. 

### 2.2. Thermal and optical analysis

**Figure 4:**
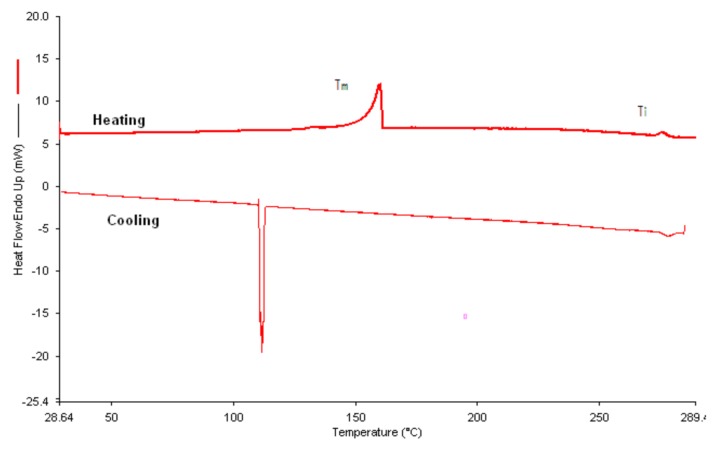
DSC thermogram of diol 2b.

**Figure 5:**
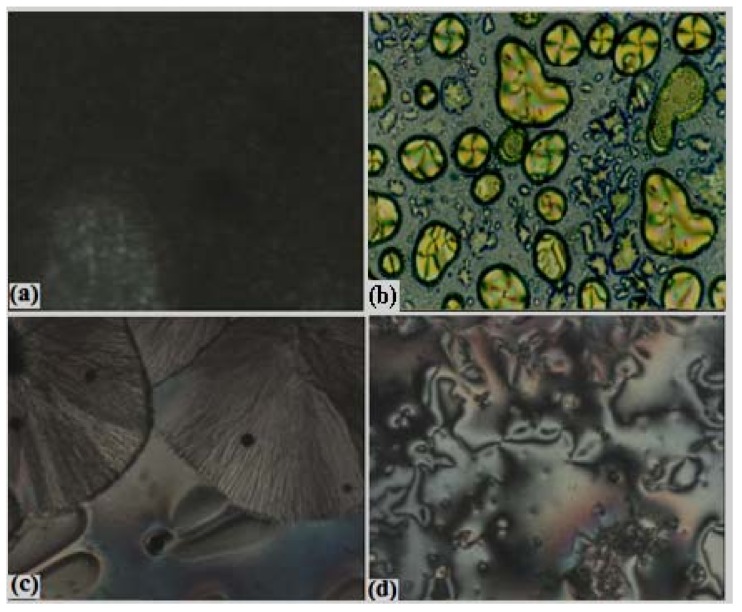
Polarized optical micrographs of (a) diol **2a**, (b) diol **2b**, (c) diol **2c**, and (d) diol **2d**.

The mesophases of the diols **2(a–d**) were studied by polarizing optical microscope (POM) and differential scanning calorimetry (DSC). Table 2 shows the mesophase confirmation and the transition temperatures for diols **2(b–d**). DSC analysis was carried out for all diols obtained and Figure 4 shows the observed transition temperature for diol **2b**. The crystal phase to nematic phase transition temperature (Cr-N) and nematic phase to isotropic phase transition (N-I) temperature is observed under POM. When the diols are observed under POM at 5 °C/min (to correlate with the DSC rate), the results revealed that the diols **2b**, and **2d** exhibit a nematic liquid crystal phase, diol **2c** exhibits a smectic liquid crystal phase, whereas diol **2a** do not show any liquid crystalline behavior, but simply changes from a solid to an isotropic fluid. Compounds that exhibit liquid crystalline behavior are due to their high aspect ratio (length to breadth ratio). The POM images for all obtained compounds are shown in Figure 5. It is worth mentioning that similar studies have been done on liquid crystals with four aromatic rings in the rigid core and when a three and four carbon alkyl chain is added to its terminal end they did not exhibit liquid crystal phases [4]. The addition of the flexible chain length decreased the melting point of these diols. This observation is supported by the previous research [10,11,12]. The lateral substituents introduced into mesogen molecules depress the thermal stability of the mesophases, it is due to the overall anisotropic broadening of the molecule will influence cooperative packing needed in the structures that form mesophase [13,14]. 

The effect of a flexible substituent in depressing the mesophase stability is greater in more ordered phases such as smectics than nematics [15]. The alkylation of the flexible chain gives a larger nematic phase range when it is added at the terminal end (located as far as possible from the rigid part of the molecule) [16]. However, the effect of dilution by adding the length of flexible chain has a limit, and further addition of the carbon chain length will increase the transition temperatures [17]. Therefore, it could be predicted that further addition of flexible chain length might increase the transition temperature point. Nevertheless, there are cases that the transition temperature will increase with increase in the alkyl chain length as reported in the works of Dave *et al*. [18]. Chemical crosslinking imposes additional constraints on the motion of chain segments which reduced the free volume that caused an increased in transition temperature [14]. Nevertheless, the clearing temperatures decrease with increasing spacer lengths too [11]. The linker group used in our present work is CH=N (an azomethine group). This linker group generally will have a higher transition temperature when compared to the ester linker group (COO). In the latter the oxygen atom of the central carbonyl group will be bumped into the non-bonded sides of the adjacent hydrogens in the aromatic ring, thereby causing considerable strain on the molecules [18]. This will cause some twist around the C-O bond and force the benzene ring with ester linkage out of the plane in the molecules. Thus reduces the coplanarity of the molecule and the broadening of rigid core. All diols used are of an even number of carbon alkyl chain length (n = 4, 6, 8, and 10) hence the odd-even effect is not shown in the melting points trend. The odd-even effect is exhibited when a subsequent increase in flexible chain length (one by one carbon atom) will result in an inconsistent increase or decrease in transition temperature. The methyl group that is bonded to each aromatic ring also plays an important role in decreasing the transition states. In this research, one of the starting materials (*o*-tolidine) possesses two methyl groups where each one is bonded to the aromatic ring in the *meta* position will increase molecular broadening of the rigid core, such that intermolecular interactions are responsible for mesomorphism decreased as the intermolecular separation increases. This would in turn increase the entropy (ΔS) of the nematic to isotropic phase transition. As explained earlier, 4-4'-di(6-hydroxyhexoxy)-*N*-benzylidene-*o*-tolidine (diol **2b**), 4-4'-di(8-hydroxyoctoxy)-*N*-benzylidene-*o*-tolidine (**2c**) and 4-4'-di(10-hydroxydecoxy)-*N,N'*-benzylidene-*o*-tolidine (**2d**), only show marbled texture when heated above its melting point. The isotropic points for diols 2(**b–d)** are 275.2 °C, 231.0 °C and 251.0 °C, respectively. Diols **2b** and **2d** exhibit typical nematic texture and no trace of smectic structure was seen in these diols. Diol **2a** do not show liquid crystalline behavior, but only melts at 242.11 °C. The reason that this compound does not exhibit any liquid crystal phase because is presumably because the carbon chain is not long enough. 

##  3. Experimental

### 3.1. Materials

Unless otherwise stated, all chemicals were commercially obtained and used without further purification or distillation: *o*-tolidine (Fluka, Germany), 4-hydroxybenzaldehyde (MERCK, Germany), 4-chloro-1-butanol (Fluka, Germany), 6-chloro-1-hexanol (Fluka, Germany), 8-chloro-1-octanol (Sigma Aldrich, Germany), 10-chloro-1-decanol (Sigma Aldrich, Germany), potassium carbonate (Fisher Chemicals, UK), absolute ethanol 99.5% (Systerm®, Malaysia), 1-butanol 99.5% (R&M Chemicals, Malaysia), *N,N*-dimethylformamide (Systerm®, Malaysia)

### 3.2. Instrumentation

FT-IR spectra were measured using a Perkin-Elmer 2000 FTIR equipped with a potassium bromide (KBr) beam splitter. Thirty-two (32) scans were collected between 4000 cm^-1^ and 400 cm^-1^ with a resolution of 2.0 cm^-1^. The ^1^H-NMR and ^13^C-NMR spectra were obtained using a Bruker 400 MHz and a 300 MHz spectrometer. The samples were prepared at 100 mg/mL in CDCl_3_ with tetramethylsilane (TMS) as the internal reference. CHN microanalyses were performed using a Perkin Elmer 2400 LS Series CHNS=O analyzer. Differential scanning calorimetry (DSC) studies were carried out with a Perkin-Elmer DSC7 series at heating rate of 5 °C/min in nitrogen, Melting points of LC monomers were obtained based on the peak of the melting temperatures from DSC analysis. Polarizing optical microscope (POM), a Carl Zeiss Axioskop equipped with 40 Linkam LTS350 hot stage, Linkam TMS94 temperature controller and Linkam LNP cooling system (pump) was used to determine the liquid crystalline mesophase. Thermal stability was studied using Perkin-Elmer TGA-7 under N_2_ atmosphere. The heating rate was programmed to 5 °C/min and it was performed at the temperature range of 30–900 °C.

### 3.3. Preparation of Bisphenol N,N'-bis(4-hydroxy)-benzylidene-o-tolidine *(**1**)*.

*p*-Hydroxybenzaldehyde (12.21 g, 0.10 mol) was added dropwise into a solution of *o*-tolidine (10.61 g, 0.05 mol) in absolute ethanol (60 mL) in a 500 mL reaction flask. The mixture was refluxed for 6 h with magnetic stirring. The product was then filtered, washed several times with diethyl ether and dried in the vacuum oven at 70 °C. Final purification was carried out by re-crystallization using 1-butanol to give yellow crystals. Yield was 74% with a melting point of 281–282 °C. FT-IR (KBr disc): 3,414 cm^-1^ (O-H stretch), 1,608 cm^-1^ (C=N stretch), 1,516 cm^-1^ (C=C stretch). ^1^H-NMR (400 MHz): δ 8.56 (s, 2H, H-C=N), 6.05 (2H, -OH), 7.79–6.85 (dd, 8H, aromatic protons), 7.35 (d, 2H, aromatic protons), 7.27 (d, 2H, aromatic protons), 7.76 (s, 2H, aromatic protons), 2.34 (s, 6H, Ph-CH_3_) ppm. ^13^C-NMR (CDCl_3_): δ 18.64, 116.21, 122.68, 126.34, 129.12, 130.62, 131.08, 132.14, 141.24, 151.36, 160.58, 161.23 ppm.

### 3.4. Preparation of mesogenic diols ***2(a–d)***

Chloroalkanol (0.40 mol) and sodium carbonate (0.45 mol) were dissolved in DMF (400 mL). Bisphenol **1** (0.20 mol) in DMF (60 mL) was added in the above mixture in a 500 mL reaction flask. The mixture was refluxed at 130 °C for 12 h. The product was precipitated by pouring the obtained solution into cold distilled water (2 L). The precipitate was then filtered, washed several times with diethyl ether and dried in a vacuum oven at 90 °C. Final purification was carried out by re-crystallization using the mixture solvent of DMF-butanol (1:1).

*4,4'-di(4-Hydroxybutoxy)-N-benzylidine-o-tolidine* (**2a**). Colour: yellow. Yield: 58%. Melting point: 242–244 °C. FT-IR (KBr disc): 3,414 cm^-1^ (O-H stretch), 1,607 cm^-1^ (C=N stretch), 1,509 cm^-1^ (C=C stretch). ^1^H-NMR (400 MHz): δ 8.37 (s, 2H, -CH=N), 7.87–6.98 (dd, 8H, aromatic protons), 7.50 (d, 2H, aromatic protons), 7.45 (d, 2H, aromatic protons), 7.85 (s, 2H, aromatic protons), 4.03 (t, 4H, -O-CH_2_-), 3.65 (m, 4H, HO-CH_2_-), 2.41 (s, 6H, Ph-CH_3_), 1.89–1.49 (m, 8H, -CH_2_- protons) ppm. ^13^C-NMR (CDCl_3_): δ 18.5 (Ph-CH_3_), 25.57–32.31 (-CH_2_-), 62.00 (-O-CH_2_-), 69.54 (-CH_2_-OH), 114.0–160.74 (aromatic carbons), 162.24 (-CH=N-) ppm. Elemental analysis: found: C, 76.43; H, 7.14; N, 5.06, C_36_H_40_N_2_O_4_.Calcd: C, 76.59; H, 7.09; N, 4.96. 

*4,4'-di(6-Hydroxyhexoxy)-N-benzylidine-o-tolidine* (**2b**). Colour: Brown. Yield: 70%. Melting point: 160–162 °C. FT-IR (KBr disc): 3,419 cm^-1^ (O-H stretch), 1,621 cm^-1^ (C=N stretch), 1,509 cm^-1^ (C=C stretch). ^1^H-NMR (400 MHz): δ 8.35 (s, 2H, -CH=N), 7.90–7.02 (dd, 8H, aromatic protons), 7.51 (d, 2H, aromatic protons), 7.49 (d, 2H, aromatic protons), 7.87 (s, 2H, aromatic protons), 4.05 (t, 4H, -O-CH_2_-), 3.65 (m, 4H, HO-CH_2_-), 2.41 (s, 6H, Ph-CH_3_), 1.90-1.30 (m, 16H, -CH_2_- protons) ppm.^13^C-NMR (CDCl_3_): δ 18.52 (Ph-CH_3_), 25.57-32.31 (-CH_2_-), 62.65 (-O-CH_2_-), 68.64 (-CH_2_-OH), 114.43–158.74 (aromatic carbons), 161.24 (-CH=N-) ppm. Elemental analysis: found: C, 77.30; H, 7.79; N, 4.50, C_40_H_48_N_2_O_4_.Calcd: C, 77.14; H, 7.74; N, 4.51. 

*4,4'-di(8-Hydroxyoctoxy)-N-benzylidine-o-tolidine* (**2c**). Colour: yellow. Yield: 68%. Melting point: 119–120 °C. FT-IR (KBr disc): 3,419 cm^-1^ (O-H stretch), 1,621 cm^-1^ (C=N stretch), 1,509 cm^-1^ (C=C stretch). ^1^H-NMR (400 MHz, CDCl_3_): δ 8.35 (s, 2H, -CH=N), 7.90–6.96 (dd, 8H, aromatic protons), 7.49 (d, 2H, aromatic protons), 7.46 (d, 2H, aromatic protons), 7.88 (s, 2H, aromatic protons), 4.02 (t, 4H, -O-CH_2_-), 3.66 (m, 4H, HO-CH_2_-), 2.42 (s, 6H, Ph-CH_3_), 1.86–1.29 (m, 24H, -CH_2_- protons) ppm. ^13^C-NMR (CDCl_3_,): δ 18.5 (Ph-CH_3_), 25.57–32.31 (-CH_2_-), 62.00 (-O-CH_2_-), 69.54 (-CH_2_-OH), 114.50–160.45 (aromatic carbons), 162.40 (-CH=N-) ppm. Elemental analysis: found: C, 78.28; H, 8.11; N, 4.20, C_44_H_56_N_2_O_4_.Calcd: C, 78.1; H, 8.28; N, 4.14. 

*4,4'-di(10-Hydroxydecoxy)-N-benzylidine-o-tolidine* (**2d**). Colour: yellow. Yield: 81%. Melting point: 108–109 °C. FT-IR (KBr disc): 3,353 cm^-1^ (O-H stretch), 2931 cm^-1^, 2,862 cm^-1^, 1,620 cm^-1^ (C=N stretch), 1509 cm^-1^ (C=C stretch). ^1^H-NMR (400 MHz, CDCl_3_): δ 8.39 (s, 2H, -CH=N), 7.92–6.98 (dd, 8H, aromatic protons), 7.51 (d, 2H, aromatic protons), 7.49 (d, 2H, aromatic protons), 7.88 (s, 2H, aromatic protons), 4.07 (t, 4H, -O-CH_2_-), 3.68 (m, 4H, HO-CH_2_-), 2.46 (s, 6H, Ph-CH_3_), 1.89–1.25 (m, 32H, -CH_2_- protons) ppm. ^13^C-NMR (CDCl_3_) δ 18.5 (Ph-CH_3_), 25.57–32.31 (-CH_2_-), 62.00 (-O-CH_2_-), 69.54 (-CH_2_-OH), 114.0–160.74 (aromatic carbons), 162.24 (-CH=N-) ppm. Elemental analysis: found: C, 78.60; H, 8.90; N, 3.80, C_48_H_64_N_2_O_4_.Calcd: C, 78.68; H, 8.74; N, 3.82.

## 4. Conclusions

A series of *N,N'*-bis(4-hydroxyl)benzylidine-*o*-tolidine compounds with various alkyl chain lengths (4, 6, 8, and 10), was successfully synthesized. The diols were found to demonstrate enantiotropic nematic phase based on the POM and DSC observations. Diols **2b** and **2d** exhibit enantiotropic nematic liquid crystal phase (marble texture and shredded texture respectively) but diol **2a** did not exhibit any liquid crystalline phase, while the diol **2c** exhibits a smectic mesophase. DSC analyses showed that the increase in alkyl chain length in diols will result in a the decrease in the transition temperature for this series of compounds. 

## Figures and Tables

**Table 1:** Physical properties and CHN data for diols **2 (a–d**).

Mesogenic diol	CHN		Color	Yield (%)	Melting Point (°C)
	Calculated (%)	Found (%)			
N,N'-bis(4-hydroxyl)benzylidine-*o*-tolidine (**1**)	C:80.0, H: 5.70, N: 6.66.	C: 79.7, H: 5.60, N: 6.56.	Yellow	88	281-282
4-4-di(4-hydroxybutoxy)-N-benzylidene-*o*-tolidine (**2a**)	C:76.59, H: 7.09, N: 4.96.	C: 76.43, H: 7.14, N: 5.06.	Yellow	58	242-244
4-4-di(6-hydroxyhexoxy)-N-benzylidene-*o*-tolidine (**2b**)	C: 77.14, H: 7.74, N: 4.51.	C: 77.30, H: 7.79, N: 4.50.	Brown	70	160-162
4-4-di(8-hydroxyoctoxy)-N-benzylidene-*o*-tolidine (**2c**)	C:78.1, H: 8.28, N: 4.14	C:78.28, H: 8.11, N: 4.20.	Yellow	68	119-120
4-4-di(10-hydroxydecoxy)-N-benzylidene-*o*-tolidine (**2d**)	C:78.68, H: 8.74, N: 3.82.	C:78.60, H: 8.90, N: 3.80.	Yellow	81	108-109

**Table 2:** Transition temperatures of diols **2 (a–d**).

Mesogenic diol	Cr-N (°C)*	N-I (°C)*	∆T/ ° C	Transition temperatures, DSC (°C)	
				Tm	Ti
4-4-di(4-hydroxybutoxy)-N-benzylidene-*o*-tolidine (**2a**)	__	__	__	242.11	__
4-4-di(6-hydroxyhexoxy)-N-benzylidene-*o*-tolidine (**2b**)	160.1	275.2	115.1	160.0	285.10
4-4-di(8-hydroxyoctoxy)-N-benzylidene-*o*-tolidine (**2c**)	126.0	231.0	105.0	130.0	228.0
4-4-di(10-hydroxydecoxy)-N-benzylidene-*o*-tolidine (**2d**)	103.0	251.0	148.0	105.0	257.0

Cr = crystal; N = Nematic phase; I = isotropic; * = as observed under POM.
